# Stimuli-Responsive Micelles with Detachable Poly(2-ethyl-2-oxazoline) Shell Based on Amphiphilic Polyurethane for Improved Intracellular Delivery of Doxorubicin

**DOI:** 10.3390/polym12112642

**Published:** 2020-11-10

**Authors:** Kang Xu, Xiaojun Liu, Leran Bu, Hena Zhang, Caihong Zhu, Yuling Li

**Affiliations:** 1 Jiangsu Key Laboratory of Green Synthetic Chemistry for Functional Materials, School of Chemistry and Materials Science, Jiangsu Normal University, Xuzhou 221116, China; wuxixukang@163.com (K.X.); xjliu@163.com (X.L.); b13585393294@163.com (L.B.); zhn8561787@163.com (H.Z.); 2 Orthopaedic Institute, Medical College, Soochow University, Suzhou 215007, China

**Keywords:** polyurethane, dual-responsive, micelles, poly(2-ethyl-2-oxazoline), doxorubicin

## Abstract

Polyurethanes (PUs) have various biomedical applications including controlled drug delivery. However, the incompletely release of drug at tumor sites limits the efficiency of these drug loaded polyurethane micelles. Here we report a novel polymer poly(2-ethyl-2-oxazoline)-SS-polyurethane-SS-poly(2-ethyl-2-oxazoline) triblock polyurethane (PEtOz-PU(PTMCSS)-PEtOz). The hydrophilic pH-responsive poly(2-ethyl-2-oxazoline) was used as an end-block to introduce pH responsiveness, and the hydrophobic PU middle-block was easily synthesized by the reaction of poly (trimethylene carbonate) diol containing disulfide bonds (PTMC-SS-PTMC diol) and bis (2-isocyanatoethyl) disulfide (CDI). PEtOz-PU(PTMCSS)-PEtOz could self-assemble to form micelles (176 nm). The drug release profile of PEtOz-PU(PTMCSS)-PEtOz micelles loaded with Doxorubicin (DOX) was studied in the presence of acetate buffer (10 mM, pH 5.0) and 10 mM dithiothreitol (DTT). The results showed that under this environment, DOX-loaded polyurethane micelles could release DOX faster and more thoroughly, about 97% of the DOX was released from the DOX-loaded PEtOz-PU(PTMCSS)-PEtOz micelle. In addition, fluorescent microscopy and cell viability assays validated that the DOX-loaded polyurethane micelle strongly inhibits the growth of C6 cells, suggesting their potential as a new nanomedicine against cancer.

## 1. Introduction

In the past few decades, biodegradable polyurethanes (PUs) have attracted growing interest because of their excellent biocompatibility and mechanical properties, and also their facile synthesis and easy introduction of functional groups. Based on these advantages, PUs have been used as important synthetic biomaterials such as drug carriers and tissue engineering. Although they have so many attractive properties, these biodegradable PUs are challenging to specifically and completely release the drug at the tumor sites [1,2]. This can lead to compromised therapeutic efficacy in vivo [3,4,5].

Recently, various stimuli-responsive polyurethane nanocarriers have been designed and developed to overcome the sluggish drug delivery and ensure the drug is delivered into the target tumor sites [3,6,7,8]. These stimuli-responsive polyurethanes were obtained in the form of biodegradable polymers containing stimuli-responsive bonds in the main chain or side chain, which can be easily destroyed in the intracellular microenvironment of the tumor, by pH [2,3,9], redox [7], temperature and so on. Among them, redox-sensitive [9,10] nanocarriers based on polyurethanes have attracted more and more attention for anticancer drug delivery [5,11,12,13]. Owing to the difference of reducing potential environment in the cytosol of cancer cells [14], these stimuli-responsive nanocarriers could be cleaved and release the drug at the same time. However, these redox-sensitive polyurethanes have got limited success in controlling the drug delivery kinetics. Incomplete release of drugs from polymer micelles is a very common problem with polymer micelles as drug carrier [15,16,17,18,19]. Aggregation of the hydrophobic cores can cause incomplete release [4]. To further improve the drug release profile, some interesting pH/redox dual-responsive drug delivery systems have been developed in recent years [3,4].

Aliphatic polycarbonate is one of the important biodegradable polymers, with good biocompatibility, excellent mechanical properties, low toxicity and biodegradability. However, one disadvantage of aliphatic ester oligomers is that acid degradation products tend to be generated during degradation, and these acid degradation products will affect the stability of the drug inside the carrier, and may even cause inflammation of tissues in the body. To overcome this problem, scientists hope to replace other aliphatic ester oligomers with low-molecular-weight polytrimethylene carbonate (PTMC). Since PTMC does not produce acidic substances after degradation, it not only eliminates the possibility of acidic degradation products affecting the drug in the polymer carrier, but also avoids tissue inflammation caused by acidic degradation products. Therefore, as a drug carrier, the hydrophobic segment PTMC has great advantages.

In recent years, poly(2-ethyl-2-oxazoline) (PEtOz) has received increasing attention as an alternative to polyethylene glycol (PEG). It could be used as an alternative to PEG for exhibiting the same “stealth” properties as PEG with a long residence time in blood and reduced accumulation in the spleen and liver [20,21]. Additionally, PEtOz can be synthesized easily by the living cationic ring-opening polymerization of 2-ethyl-2-oxazoline and has been approved by the US Food and Drug Administration as a food additive [18,22,23]. PEtOz is a pH-sensitive polymer with good biocompatibility and biodegradability, which can be protonated in the low pH environment of the lysosome [24,25]. This property of PEtOz promotes the drug release of micelles with PEtOz as the hydrophilic shell.

In current work, we design and prepare a new amphiphilic triblock PUs, poly(2-ethyl-2-oxazoline)-*block*-polyurethane-*block*-poly(2-ethyl-2-oxazoline) (PEtOz-*b*-PU(PTMCSS)-*b*-PEtOz), which can self-assembled into a pH and reduction dual-sensitive shell-detachable micelle for delivery of antitumor drugs to C6 cell. This drug delivery system based on triblock polyurethanes has the following features [26]. (i) Easy and facile synthesis of triblock PUs. Compared with the traditional method, the synthesis of PEtOz-*b*-PU(PTMCSS)-*b*-PEtOz provides a simple one pot multistep method, which involved easy separation and purification of polymer, also mean the possibility of preparation of PUs in large scale. (ii) The PEtOz segment in triblock polymer forms the shell of micelle and provides some excellent properties. One is the stealth-like property, which is similar to PEG during its longer circulation [12,20]. The other is pH sensitivity, which can destabilize in endosomes of cancer cells [27]. (iii) The disulfide-bridged of core made of hydrophobic polyurethane will be cleaved in the intracellular cytosol of the cancer cells due to the high glutathione (GSH) concentration (2–10 mM) [28]. (iv) The system uses low-molecular-weight PTMC to replace other aliphatic ester oligomers. It not only eliminates the possibility of acid degradation products affecting the drug in the polymer carrier, but also avoids tissue inflammation that may be caused by acid degradation products.

To our knowledge, little work has been done using PEtOz as the hydrophilic segment of PUs [29,30,31,32,33]. This polymer is expected to further improve the biocompatibility and biodegradability of PUs, and expand its application in the field of biomedicine.

## 2. Materials and Methods

### 2.1. Materials

Bis (2-isocyanatoethyl) disulfide (CDI) and 1,6-diisocyanatohexane (HDI) were supplied by energy-chemical. Trimethylene carbonate (TMC), methyl tosylate (MeOTs), stannous octoate (Sn(Oct)_2_) and 2-ethyl-2-oxazoline (EtOz, 99%) were obtained from Alfa Aesar (Shanghai, China). The potassium hydroxide, ether, toluene, dichloromethane (DCM) and *N*,*N*-dimethylformamide (DMF) was obtained from Sinopharm Chemical Reagent Co., Ltd. (Shanghai, China). Dithiothreitol (DTT) was purchased from Dongren Chemical Technology Co., Ltd. (Suzhou, China). Doxorubicin hydrochloride (DOX HCl) was provided by Beijing ZhongShuo Pharmaceutical Technology Development Co., Ltd. (Beijing, China). While the RPMI-1640, fetal bovine serum (FBS, Gibco), and 96-well plates were supplied by Corning Costar (Shanghai, China). 3-(4,5-dimethyl-2-thiazolyl)-2,5-diphenyl-2*H*-tetrazolium bromide (MTT) was provided by Biosharp. 4′,6-diamidino-2-phenylindole (DAPI) was purchased from Roche. All other chemicals were of analytical grade or better.

### 2.2. Instruments and Measurements

The ^1^H NMR (400 MHz) was recorded using a Bruker 400 MHz apparatus (Bruker, Billerica, MA, USA). The molecular weight (*M*w) and polydispersity index (PDI) of the polymers measured by using a PL GPC 50 instrument (Agilent, Santa Barbara, CA, USA) equipped with Jordi GPC columns (10E4, 2M) following a differential refractive-index detector (PL-RI). The measurements were performed using HPLC-grade DMF containing 0.1 N LiBr as the eluent at a flow rate of 1.0 mL/min at 50 °C and a series of narrow polystyrene standards for the calibration of the columns.

The size of micelle was measured with dynamic light scattering (DLS) using a Nano-ZS from Malvern Instruments (Malvern, Malvern, UK). The block copolymer micelle solutions were filtered through a 0.45-μM filter prior to use. The particle morphology was analyzed by a S-8010 scanning electron microscope (SEM) (Hitachi, Tokyo, Japan). The SEM samples were prepared on monocrystalline silicon wafer. The micelle solutions were then floated on smooth surface of silicon wafer. The cellular uptake and intracellular release behaviors of DOX-loaded PEtOz-PU(PTMCSS)-PEtOz micelles was observed by fluorescence microscope (Nikon eclipse Ts2R, Tokyo, Japan). Optical density (OD) values for each well in the assay were measured using a thermo scientific multiskan go microplate reader in MTT experiments. The fluorescence of doxorubicin was measured by F-4500 FL Spectrophotometer (Hitachi, Tokyo, Japan) at 298 K.

### 2.3. Synthesis of PEtOz-OH

PEOtz-OH was synthesized by the cationic ring-opening polymerization of 2-ethyl-2-oxazoline (EOz) using methyl p-toluenesulfonate (MeOTs) as an initiator, as reported previously [34]. Briefly, a solution of EOz (10 g, 0.101 mol) and MeOTs (0.34 g, 1.834 × 10^−3^ mol) in acetonitrile (33.3 mL) were added to the predried reaction flask, and then stirred at reflux (100 °C) in an oil bath for 24 h under nitrogen. After cooling to room temperature, the resulting product was added to 0.1 M of methanolic KOH and the reaction was maintained for 4 h to introduce hydroxyl groups at the end of the PEOz chain, then settled with ice ether, centrifuged to collect the solid, and dried in vacuum for 24 h. Yield: 83%.

### 2.4. Synthesis of PTMC-SS-PTMC

Under the protection of nitrogen, 60 mL of toluene, dithioethylene glycol (HES) (1.61 g, 10.48 mmol), and TMC monomer (10.71 g, 104.80 mmol) were added into a closed three-neck flask with a stir bar, and then react at 100 °C for 24 h. After that, the reaction solution was cooled to room temperature, and a few drops of concentrated hydrochloric acid were added and stirred for 30 min to terminate the reaction. Finally, the reaction solution was concentrated and settled in ice ether, and the precipitate was collected by centrifugation and dried in vacuum for 24 h to obtain PTMC-SS-PTMC. Yield: 87%.

### 2.5. Synthesis of PEtOz-PU(PTMCSS)-PEtOz

**Scheme 1:**
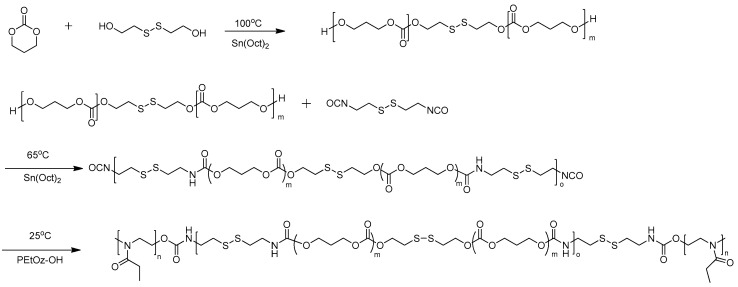
Synthesis of poly(2-ethyl-2-oxazoline)-*block*-polyurethane-*block*-poly(2-ethyl-2-oxazoline) (PEtOz-PU(PTMCSS)-PEtOz) copolymer.

The polyurethane PEtOz-PU(PTMCSS)-PEtOz was synthesized based on PEOtz-OH, PTMC-SS-PTMC diols and CDI. The reaction scheme is briefly described in Scheme 1. Briefly, in a dry nitrogen atmosphere, the PTMC-SS-PTMC diol (3.00 g, 3.00 mmol) and a catalytic amount of Sn(Oct)_2_ were first dissolved in DMF at 65 °C. Then, the specified amount of CDI (0.67 g, 3.30 mmol) was added and stirred at 65 °C for 24 h. After that, in a nitrogen atmosphere and a low temperature, the PEtOz-OH solution dissolved in DMF was added dropwise, and then the reaction was continued at 25 °C for 48 h. Lastly, the flask was cooled to room temperature. After the reaction was completed, the mixture was added dropwise to an excess of diethyl ether to obtain the resulting copolymer, and purified by repeated precipitation in an diethyl ether-methanol solution, and eventually vacuum-dried at room temperature for 48 h. The yield of the resulting copolymer was 74%, and the synthesis of PEtOz-PU(PTMC)-PEtOz was similar with the synthesis of PEtOz-PU(PTMCSS)-PEtOz. The yield of the resulting copolymer was 67%.

### 2.6. Preparation and Characterization of PEtOz-PU(PTMCSS)-PEtOz Polymer Micelles

The PEtOz-PU(PTMCSS)-PEtOz micelles were fabricated by using dialysis method. Two milligrams of PEtOz-PU(PTMCSS)-PEtOz were dissolved in 1 mL of DMSO, and then 1.5 mL of distilled water was added dropwise. The resulting solution was transferred to a dialysis membrane (MWCO 3500) and dialyzed with distilled water at 25 °C. After 24 h, the solution was removed from the membrane and the polymer had been assembled into micelles. The solution was then filtered through a 0.45 μm filter and lyophilized in a freeze dryer. The critical micelle concentration (CMC) of PEtOz-PU(PTMCSS)-PEtOz micelle was determined by fluorescence method using pyrene as probe. The combined solution of pyrene and micelles was performed by F-4500 FL Spectrophotometer at a wavelength of 340 to 500 nm with the excitation wavelength of 330 nm. The in vitro stability, pH and reduction-responsiveness of PEtOz-PU(PTMCSS)-PEtOz were also studied by DLS.

In order to obtain DOX-loaded PEtOz-PU(PTMCSS)-PEtOz micelles, PEtOz-PU(PTMCSS)-PEtOz (5 mg) and DOX were completely dissolved in 2 mL DMSO and sonicated for 0.5 h. Then, 3 mL of water was slowly added to the resulting solution under vigorous stirring and sonicated for 1 h. Subsequently, the resulting solution was transferred to a dialysis membrane (MWCO 3500 Da) and dialyzed against distilled water for 24 h to obtain drug-loaded micelles.

In order to quantitively analyze drug encapsulation, the equal samples of the lyophilized drug-carrying micelle solution were lyophilized, dissolved in 2 mL DMSO, and then analyzed by fluorescence spectroscopy. The characteristic absorbance of DOX (560 nm) was recorded and compared to a ten-point standard curve of DOX (0–250 mg/μL in DMSO). The percentage of drug loading content (DLC) and drug loading efficiency (DLE) were calculated using Equations (1) and (2):

DLC (wt.%) = (weight of loaded drug/total weight of loaded drug and polymer) × 100(1)

DLE (%) = (weight of loaded drug/weight of drug in feed) × 100(2)

For the in vitro drug release studies, a specified amount of the DOX-loaded micelle solution was prepared in the dialysis membrane (MWCO 12,000–14,000 Da) and the drug-released were performed in the following conditions, (i) released in PBS (10 mM, pH 7.4), (ii) released in acetate buffer (10 mM, pH 5.0), (iii) released in PBS containing 10 mM DTT (10 mM, pH 7.4) and (iv) in acetate buffer containing 10 mM DTT (10 mM, pH 5.0). Then, the samples were placed in a 37 °C thermostatic shaker (200 rpm). At the planned time, 4 mL of the releasing medium was removed and an equal volume of fresh releasing medium was added. After sampling, the amount of DOX released was measured by an F-4600 fluorescence spectrophotometer.

### 2.7. Cell Viability Assay

The cytotoxicity of PEtOz-PU(PTMCSS)-PEtOz micelles was measured by the MTT method. The cells we used were C6 cell lines. First, C6 cells were plated in a 96-well plate at a density of 5 × 10^3^ cells/well and placed at 37 °C for 24 h. The medium was then replaced with 100 μL medium containing PEtOz-PU(PTMCSS)-PEtOz and PEtOz-PU(PTMC)-PEtOz micelles and incubated for 24 h at 37 °C. After incubation, 20 μL of MTT reagent was added to each well. After 4 h, the medium was aspirated out and MTT-formazan was dissolved in 150 μL DMSO. Then the cell viability was determined by measuring the absorbance at 490 nm using a thermo scientific multiskan go microplate reader.

The in vitro cytotoxicity of DOX-loaded PEtOz-PU(PTMCSS)-PEtOz and PEtOz-PU(PTMC)-PEtOz micelles was evaluated by the similar method as PEtOz-PU(PTMCSS)-PEtOz and PEtOz-PU(PTMC)-PEtOz micelles, and the final concentrations of DOX in the well were varied from 0.01, 0.1, 0.5, 1, 5, 10, 20 to 40 μg/mL.

### 2.8. In Vitro Cellular Uptake and Intracellular Release

Briefly, the C6 cells (1 × 10^5^ cells/well) were seeded in six-well plates. After 24 h, the C6 cells were incubated with the DOX-loaded PEtOz-PU(PTMCSS)-PEtOz and PEtOz-PU(PTMC)-PEtOz micelles in the dark for 2 h and 4 h at 37 °C, respectively. Then, the medium was removed and the cells were washed three times with PBS. After that, the cells were fixed with 4% paraformaldehyde. Then cells were stained with DAPI for 20 min and washed three times with PBS. Finally, the fluorescence was visualized using fluorescence microscope.

## 3. Results and Discussion

### 3.1. Synthesis of Polyurethane (PU) Copolymers

PEtOz-PU(PTMCSS)-PEtOz was prepared by the polycondensation of PTMC-SS-PTMC diols and CDI, followed by end-capping with PEtOz-OH. PTMC-SS-PTMC diols and PEtOz-OH were characterized by ^1^H NMR and GPC (Figure S1). During this process, the polycondensation between CDI and PTMC-SS-PTMC diols (*M*_n_ = 1000) was carried out at an feeding molar ratio of 1.1:1 in DMF by using Sn(Oct)_2_ as catalyst, then PEtOz-OH (*M*_n_ = 5000) was added to the above mixture and reacted for another 48 h to obtain the resulting polymer with a yield of 74%. We used ^1^H NMR spectroscopy and GPC measurement to analyze the structure of triblock polyurethane PEtOz-PU(PTMCSS)-PetOz, as summarized in Table S1. Another triblock polymer PEtOz-PU(PTMC)-PEtOz was synthesized as a control using similar method, that is, using PTMC diols and HDI to substitute for PTMC-SS-PTMC diols and CDI, respectively.

**Figure 1:**
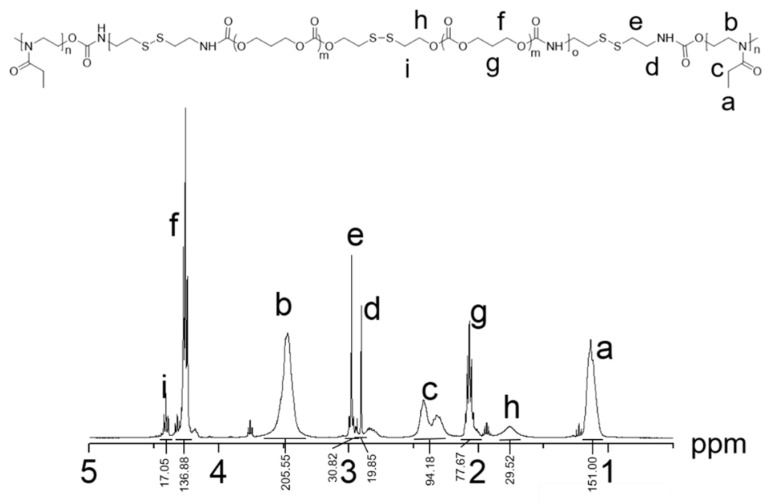
^1^H NMR spectra (400 MHz, C*D*Cl_3_) of PEtOz-PU(PTMCSS)-PEtOz.

The ^1^H NMR spectra of PEtOz-PU(PTMCSS)-PEtOz (Figure 1) showed the characteristic peaks of PEtOz moieties at δ1.096, 3.448, 2.287 ppm, the peaks of PTMC-SS-PTMC moieties at δ1.722, 2.047, 2.063, 4.234, 4.367 ppm, and the peaks of CDI moieties at δ2.880 and 2.955 ppm. As we can see in Figure S1, GPC curve of polymer PEtOz-PU(PTMCSS)-PEtOz had a unimodal distribution with a low PDI value of 1.17, which means that the synthesized polyurethanes had a uniform molecular weight with a value of 25.6 kg/mol. End group analysis of ^1^H NMR showed that the *M*_n_ value of PEtOz-PU(PTMCSS)-PEtOz is 21.0 kg/mol (Table S1). The difference of molecular weight between GPC measurement and ^1^H NMR measurement is that the standard sample in GPC measurement is different from the polyurethane we synthesized. These results clearly demonstrate that we have successfully synthesized triblock polymer PEtOz-PU(PTMCSS)-PEtOz.

### 3.2. Fabrication and Characterization of PEtOz-PU(PTMCSS)-PEtOz Micelles

Amphiphilic polymer PEtOz-PU(PTMCSS)-PEtOz could self-assemble to form micelles. A simple method of solvent exchange was adopted to prepare PEtOz-PU(PTMCSS)-PEtOz micelles. DLS results showed that the resulting PEtOz-PU(PTMCSS)-PEtOz micelles possess a size of 175.9 ± 6.6 nm and a narrower distribution with PDI of 0.11 (Figure S2). Scanning electron microscope (SEM) images of PEtOz-PU(PTMCSS)-PEtOz micelles revealed that these micelles had a mean diameter of 180 nm, which was close to the DLS results. These results proved that polymer PEtOz-PU(PTMCSS)-PEtOz was successfully assembled into uniform micelles in water. The CMC of PEtOz-PU(PTMCSS)-PEtOz micelles was determined using pyrene as a probe. As shown in Table 1 and Figure S3, PEtOz-PU(PTMCSS)-PEtOz micelles had a lower CMC of 0.43 mg/L. This result revealed that PEtOz-PU(PTMCSS)-PEtOz micelles can keep stable even at a very low concentration, leading to excellent stability and long circulation in body fluids.

In 2019, our research group reported a shell separable micelle with pH and redox sensitivity based on amphiphilic triblock polyoxazoline-polycaprolactone polyurethane-polyoxazoline (PEtOz-*b*-PU(SS)-*b*-PEtOz) for the delivery of anticancer drugs [33]. Compared with PEtOz-*b*-PU(SS)-*b*-PEtOz micelles, PEtOz-PU(SS)-PEtOz has similar size and smaller CMC. This helps the micelles maintain better stability in the body, and the morphology of the micelles can be maintained at very low concentrations.

### 3.3. Loading and Triggered Release of DOX

DOX was chosen as a model drug to explore the drug loading efficiency and drug delivery behaviours of PU micelles. Moreover, the micelles were prepared via a solvent exchange method. DOX was loaded into PEtOz-PU(PTMCSS)-PEtOz micelles at theoretical DLC of 10 and 20 wt.%, respectively. DLC and DLE was calculated according to the standard curve of DOX and results were listed in Table S2. We can see from Table S2 that DLE of DOX-loaded PEtOz-PU(PTMCSS)-PEtOz micelles ranged from 40.0 to 62.5% and practical DLC ranged from 6.3 to 8.0 wt.% could be achieved respectively.

Compared with blank micelle of PEtOz-PU(PTMCSS)-PEtOz, the average diameter of DOX-loaded PEtOz-PU(PTMCSS)-PEtOz micelles were slightly increased after drug loading, while distribution had no obvious change. The drug-loaded micelles in the range of such size can be easily phagocytosed by the tumor cells, resulting in beneficial transportion of DOX into tumor cells.

**Figure 2:**
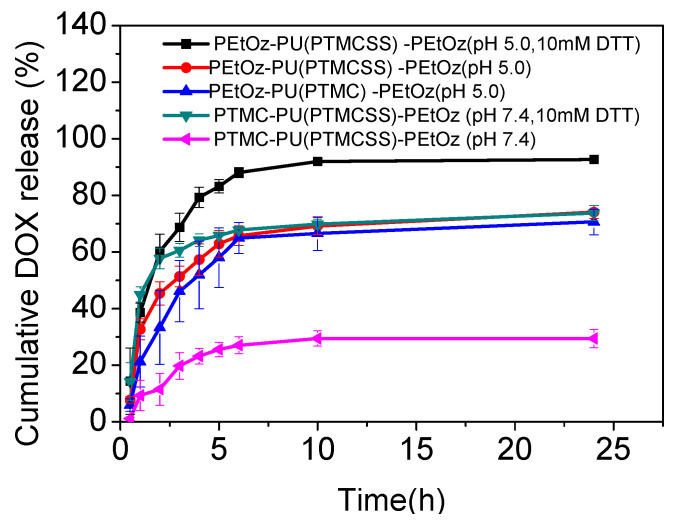
Reduction and/or pH triggered drug release from DOX-loaded PEtOz-PU(PTMCSS) -PEtOz micelles. PEtOz- PU(PTMC)-PEtOz micelles were used as a control.

We used DOX-loaded PEtOz-PU (PTMC) -PEtOz micelles as a control group to conduct in vitro drug release studies under different conditions that mimic intracellular conditions. As shown in Figure 2, compared with other groups, DOX-loaded PEtOz-PU(PTMCSS)-PEtOz micelles in response to the environment of acetate buffer (10 mM, pH 5.0) containing 10 mM DTT got the maximum release; ca. 79% and 92% of DOX were released in 4 h and 24 h, respectively. This is similar to the 76% and 95% of the PEtOz-PU(SS)-PEtOz micelles we have previously reported, and both have excellent rapid drug release capabilities in tumors. In contrast, only 73% of drug was released in PBS containing 10 mM DTT within 24 h and 74% of DOX was released in acetate buffer (10 mM, pH 5.0) within 24 h. Only 29% of the loaded drug was released within 24 h in PBS (10 mM, pH 7.4). What’s more, only 70% of the loaded drug was released from DOX-loaded PEtOz-PU(PTMC)-PEtOz micelles within 24 h in acetate buffer (10 mM, pH 5.0). From these results, we can conclude that the increase of DOX release is due to the structure of the polyurethane micelle: the pH-sensitive hydrophilic PEtOz and the disulfide bonds in the main chain of amphiphilic polyurethane. Moreover, the cleavage of the disulfide bridge between the hydrophilic and hydrophobic polymer blocks could further enhance release of DOX from drug-loaded PEtOz-PU(PTMCSS)-PEtOz micelles (Figure 2). These results indicate that the DOX-loaded PEtOz-PU(PTMCSS)-PEtOz micelles we prepared have dual redox and pH stimulus and induced synergistic drug releasing.

### 3.4. In Vitro Cytotoxicity of the PEtOz-PU(PTMCSS)-PEtOz Micelles and DOX-Loaded PEtOz-PU(PTMCSS)-PEtOz Micelles

**Figure 3:**
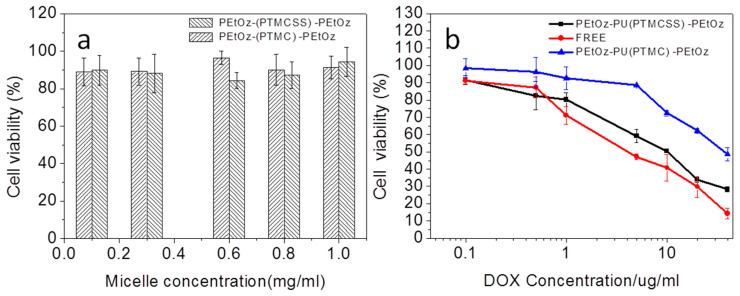
(**a**) Cytotoxicity of different concentration of PEtOz-PU(PTMCSS)-PEtOz and PEtOz-PU(PTMC)-PEtOz micelles, against C6 cells after 24 h incubation using 3-(4,5-dimethyl-2-thiazolyl)-2,5-diphenyl-2*H*-tetrazolium bromide (MTT) assay. (**b**) Anti-tumor activity of DOX-loaded PEtOz-PU(PTMCSS)-PEtOz micelles in C6 cells. DOX-loaded PEtOz-PU(PTMC)-PEtOz micelles were used as controls. The cells were treated with DOX-loaded micelles or free DOX for 48 h. Data are presented as mean ± SD (*n* = 6).

The cytotoxicity of PEtOz-PU(PTMCSS)-PEtOz micelles was evaluated and antitumor activity of the DOX-loaded PEtOz-PU(PTMCSS)-PEtOz micelles were performed in C6 cells by MTT assays. We used various concentrations of PEtOz-PU(PTMCSS)-PEtOz micelles from 0.1 mg/mL to 1.0 mg/mL to treat C6 cells. It can be seen from Figure 3a that even at a high concentration of 1.0 mg/mL for 48 h, PEtOz-PU(PTMCSS)-PEtOz micelles have almost no significant toxic effect on C6 cells. These results confirmed that PEtOz-PU(PTMCSS)-PEtOz micelles have good cytocompatibility.

As shown in Figure 3b, DOX-loaded PEtOz-PU(PTMCSS)-PEtOz micelles caused obvious growth inhibition of C6 cells, with the IC50 reached 8.4 μg DOX equiv./mL in C6 cells. In contrast, C6 cells treated by various concentrations of the DOX-loaded PEtOz-PU(PTMC)-PEtOz showed lower growth inhibition (IC50 = 46.6 μg/mL). For DOX-loaded PEtOz-PU(PTMCSS)-PEtOz micelles, lower cell viability demonstrates that there was a greater intracellular release of DOX from the PEtOz-PU(PTMCSS)-PEtOz micelles in C6 cells. This further demonstrated that PEtOz-PU(PTMCSS)-PEtOz micelles have the reduction- responsiveness because of the disulfide bonds contained in the main chain of polymer PEtOz-PU(PTMCSS)-PEtOz. From these results, we can also see that the IC50 of PUs was higher than free DOX·HCl (IC50 = 4.5 μg/mL), the reduced toxicity of DOX-loaded PEtOz-PU(PTMCSS)-PEtOz micelles is likely due to the protection of polyoxazoline and inefficient cellular uptake. These results are consistent with the PEtOz-b-PU(SS)-b-PEtOz results we have previously reported, and PEtOz-PU(PTMCSS)-PEtOz drug-loaded micelles have a lower 50% inhibitory concentration (IC50) than PEtOz-b-PU(SS)-b-PEtOz, which is more conducive to the treatment of tumors.

### 3.5. Cellular Uptake of the DOX-Loaded PEtOz-PU(PTMCSS)-PEtOz Micelles and Intracellular Tracking

**Figure 4:**
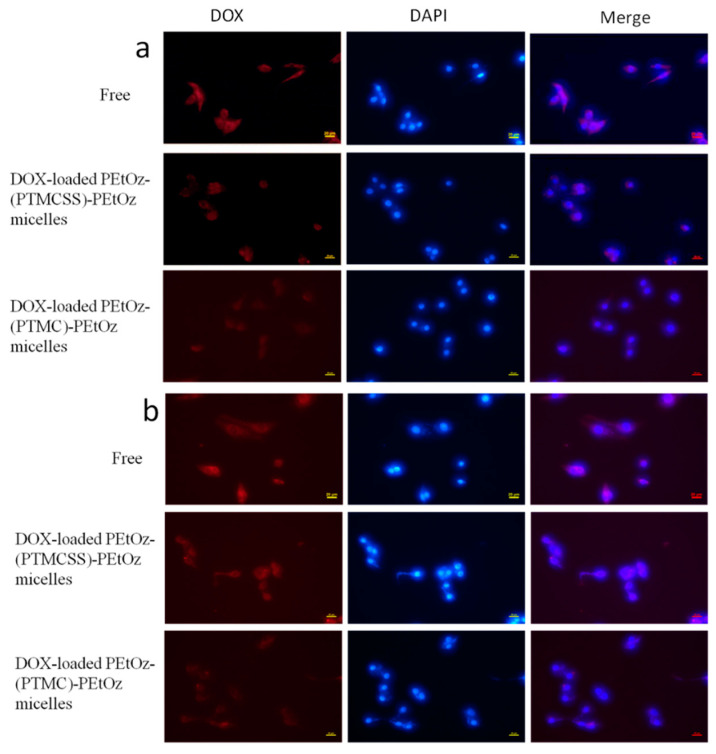
(**a**) Fluorescence microscope images of C6 cells incubated with DOX-loaded PEtOz-PU(PTMCSS)-PEtOz micelles, DOX-loaded PEtOz-PU(PTMC)-PEtOz micelles and free DOX (10 μg/mL) in 2 h. (**b**) Fluorescence microscope images of C6 cells incubated with DOX-loaded PEtOz-PU(PTMCSS)-PEtOz micelles, DOX-loaded PEtOz-PU(PTMC)-PEtOz micelles and free DOX (10 μg/mL) in 4 h. For each panel, images from left to right show DOX fluorescence in cells (red), cell nuclei stained by 2-(4-Amidinophenyl)-6-indolecarbamidine dihydrochloride (DAPI, blue) and overlays of two images. The scale bars correspond to 20 μm in all the images.

Fluorescence microscopy was used to visualize the cellular uptake and intracellular release of DOX from the DOX-loaded PEtOz-PU(PTMCSS)-PEtOz micelles in C6 cell lines. It is worth noting that the process by which DOX enters the intracellular environment (the endosome, lysosome, and cytosol) is essential for its penetration into the nucleus and subsequent interaction, as DOX induces cell death through interaction with DNA [35,36]. As shown in Figure 4, a stronger fluorescence is observed in the perinuclear region of the C6 cells after incubation with DOX-loaded PEtOz-PU(PTMCSS)-PEtOz micelles for 2 h, and even stronger DOX fluorescence was observed in the cytoplasm and perinuclei regions of C6 cells following 4 h incubation with DOX-loaded PEtOz-PU(PTMCSS)-PEtOz micelles, corroborating a high intracellular free DOX level. In contrast, after 4 h of treatment with DOX-loaded PEtOz-PU(PTMC)-PEtOz micelles, almost no DOX fluorescence was observed in C6 cells. These results were consistent with the drug release and in vitro cytotoxicity experiments. Therefore, we concluded that DOX-loaded PEtOz-PU(PTMCSS)-PEtOz micelles showed an excellent and efficient release of DOX via pH-induced and disulfide-induced destabilization of micelle.

## 4. Conclusions

In this work, a novel pH and redox dual stimuli-responsive PEtOz-PU(PTMCSS)-PEtOz micelles were prepared for tumor-triggered drug delivery in tumor cell. On the one hand, the PEtOz of amphiphilic PEtOz-PU(PTMCSS)-PEtOz maintained the “stable” state under neutral pH conditions while the PEtOz shell could swell under the acidic condition. This destabilized pH-responsive shell enhanced the triggered release of DOX into the cancer cells. On the other hand, DOX-loaded PEtOz-PU(PTMCSS)-PEtOz micelles were degraded and released most of the loaded DOX in reducing environment when disulfide bonds on the main chain of polymer broken. In vitro drug release profiles and cell experiments also confirmed that the PEtOz-PU(PTMCSS)-PEtOz micelles caused controlled DOX release to C6 cells, which indicates these biodegradable polyurethane micelles appear to be an appealing platform for triggered intracellular anticancer drug delivery.

## Figures and Tables

**Table 1:** Characteristics of PEtOz-PU(PTMCSS)-PEtOz and PEtOz-PU(PTMC)-PEtOz micelles.

Block Copolymers	Micelles Size (nm) ^a^	PDI ^a^	Zeta (mv) ^a^	CMC (mg/L) ^b^
PEtOz-PU(PTMCSS)-PEtOz	175.9 ± 6.6	0.11	−20.6	0.43
PEtOz-PU(PTMC)-PEtOz	144.6 ± 3.8	0.20	−23.4	2.25

**^a^** Measured by dynamic light scattering (DLS) at 25 °C; **^b^** measured by fluorescence using pyrene as hydrophobic probe (pyrene final concentration is 0.6 μM).

## Supplementary Materials

The following are available online at https://www.mdpi.com/2073-4360/12/11/2642/s1, Figure S1: (a) ^1^H NMR spectra (400 MHz, CDCl_3_) of PEtOz-OH. (b) ^1^H NMR spectra (400 MHz, CDCl_3_) of PTMC-SS-PTMC. (c) ^1^H NMR spectra (400 MHz, CDCl_3_) of PEtOz-PU(PTMC)-PEtOz. (d) GPC of PEtOz-PU(PTMCSS)-PEtOz, PEtOz-PU(PTMC)-PEtOz, PEtOz-OH and PTMC-SS-PTMC diol (Polystyrenestandard, eluant: DMF, temperature: 50 °C, RI detection). Figure S2: (a) Size distribution of PEtOz-PU(PTMCSS)-PEtOz micelles and PEtOz-PU(PTMC)-PEtOz micelles. (b) SEM image of PEtOz-PU(PTMCSS)-PEtOz micelles. (c) SEM image of PEtOz-PU(PTMC)-PEtOz micelles. Figure S3: Fluorescence intensity ratio I_1_/I_3_ of pyrene as a function of PEtOz-PU(PTMCSS)-PEtOz micelles and PEtOz-PU(PTMCSS)-PEtOz micelles concentration ((a) PEtOz-PU(PTMCSS)-PEtOz micelles, (b) PEtOz-PU(PTMC)-PEtOz micelles). Table S1: Characteristics of PEtOz-PU(PTMCSS)-PEtOz and PEtOz-PU(PTMC)-PEtOz block copolymers. Table S2: Drug loading content and drug loading efficiency of DOX-loaded micelles of PEtOz-PU(PTMCSS)-PEtOz and PEtOz-PU(PTMC)-PEtOz.
