# Supplementary material for: Stimuli-Responsive Micelles with Detachable Poly(2-ethyl-2-oxazoline) Shell Based on Amphiphilic Polyurethane for Improved Intracellular Delivery of Doxorubicin
Source: Polymers (Basel). 2020 Nov 10;12(11):2642. doi: 10.3390/polym12112642 (PMC7696422; doi:10.3390/polym12112642)
Supplement: Supplementary file 1 [file polymers-12-02642-s001.pdf]

polymers

MDPI

Supplementary information

Stimuli-Responsive Micelles with Detachable Poly (2-ethyl-2-oxazoline) Shell Based on Amphiphilic Polyurethane for Improved Intracellular Delivery of Doxorubicin

Kang Xu[1,†], Xiaojun Liu[1,†], Leran Bu[1], Hena Zhang[1], Caihong Zhu[2,*], Yuling Li[1,*]

[1] School of Chemistry and Materials Science, Jiangsu Key Laboratory of Green Synthetic Chemistry for Functional Materials, Jiangsu Normal University, Xuzhou 221116, China
[2] Orthopaedic Institute, Medical College, Soochow University, Suzhou 215007, China
* Correspondence: zhucaihong@suda.edu.cn (C.Z.); Ylli19722@163.com (Y.L.); Tel.: +86-516-83403165 (Y.L.)
† These authors contributed equally to this work.

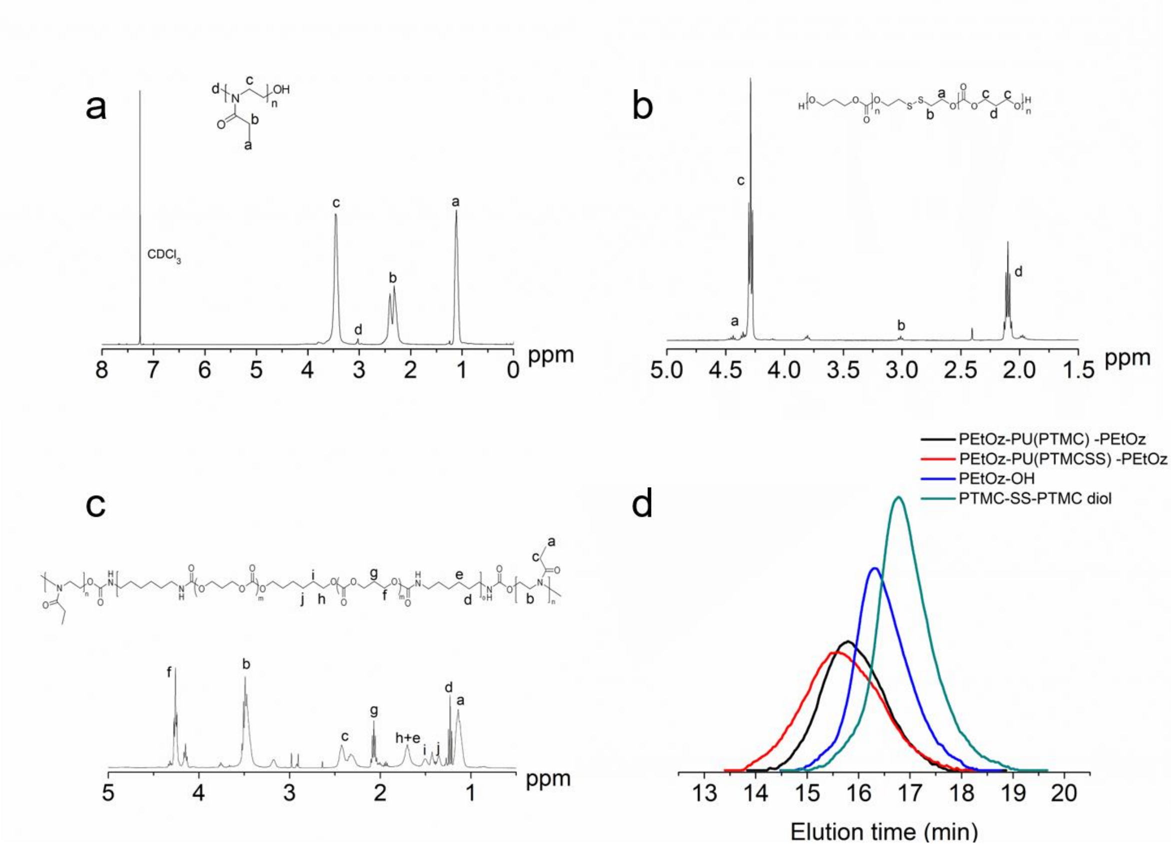

Figure S1. (a) ^{1}H NMR spectra (400 MHz, CDCl$_3$) of PEtOz-OH. (b) ^{1}H NMR spectra (400 MHz, CDCl$_3$) of PTMC-SS-PTMC. (c) ^{1}H NMR spectra (400 MHz, CDCl$_3$) of PEtOz-PU(PTMC)-PEtOz. (d) GPC of PEtOz-PU(PTMCSS)-PEtOz, PEtOz-PU(PTMC)-PEtOz, PEtOz-OH and PTMC-SS-PTMC diol (Polystyrenestandard, eluant: DMF, temperature: 50 °C, RI detection).

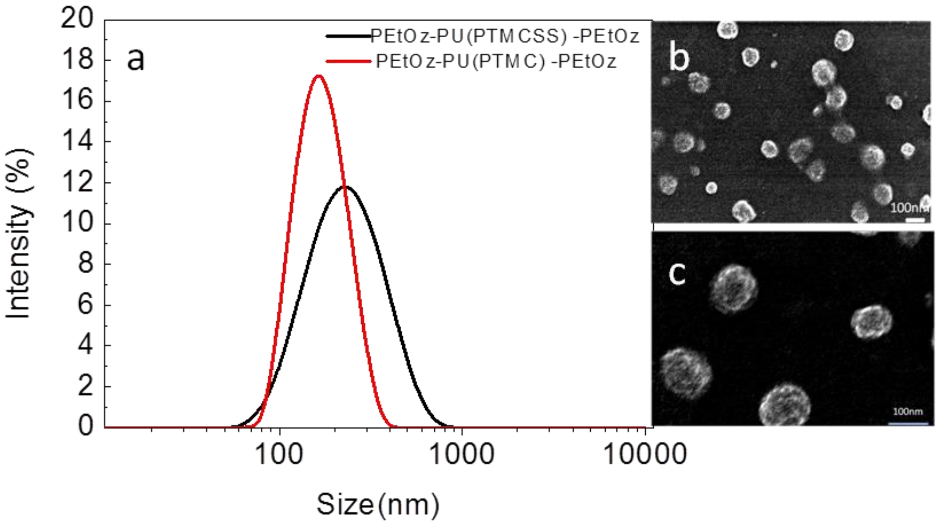

Figure S2. (a) The size distribution of PEtOz-PU(PTMCSS)-PEtOz micelles and PEtOz-PU(PTMC)-PEtOz micelles. (b) SEM image of PEtOz-PU(PTMCSS)-PEtOz micelles. (c) SEM image of PEtOz-PU(PTMC)-PEtOz micelles.

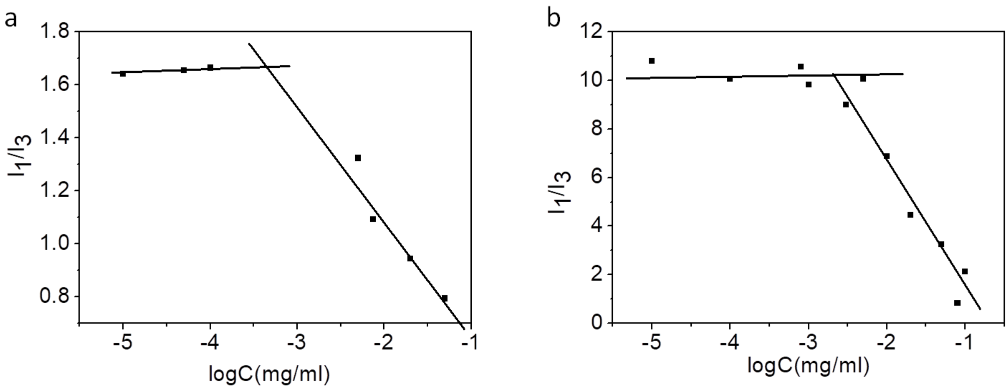

Figure S3. The fluorescence intensity ratio I_1/I_3 of pyrene as a function of PEtOz-PU(PTMCSS)-PEtOz micelles and PEtOz-PU(PTMCSS)-PEtOz micelles concentration ((a) PEtOz-PU(PTMCSS)-PEtOz micelles, (b) PEtOz-PU(PTMC)-PEtOz micelles).

Table S1. Characteristics of PEtOz-PU(PTMCSS)-PEtOz and PEtOz-PU(PTMC)-PEtOz block copolymers.

**Block Copolymers**	**Mn (^{1}H NMR) [a]**	**Mn (GPC) [b]**	**PDI (GPC) [b]**
PEtOz-PU(PTMCSS) -PEtOz	21000	25600	1.17
PEtOz-PU(PTMC) -PEtOz	17800	21300	1.11
PEtOz-OH	5000	7300	1.07
PTMC-SS-PTMC	1000	3200	1.09

[a] determined by ^{1}H NMR (400 MHz, $CDCl_3$) ;

[b] determined by GPC (DMF as the eluent, 1.0 mL/min, 50 °C, polystyrene standards).

Table S2. Drug loading content and drug loading efficiency of DOX loaded micelles of PEtOz-PU(PTMCSS)-PEtOz and PEtOz-PU(PTMC)-PEtOz.

Sample	Theoretical drug loading content wt. %	DLE %[b]	DLC Wt. %[b]	Micelle Size[a] (nm)	PDI[a]
PEtOz- PU(PTMCSS)-PEtOz	10%	62.5	6.3	183.7±3.8	0.03
	20%	40.0	8.0	214.3±3.9	0.15
PEtOz- PU(PTMC)-PEtOz	10%	54.5	5.5	161.1±1.7	0.13
	20%	42.5	8.5	238.4±5.4	0.19

[a] measured by DLS at 25 °C;

[b] DLE and DLC was determined by fluorescence measurements.
